# Persistency, medication prescribing patterns, and medical resource use associated with multiple sclerosis patients receiving oral disease-modifying therapies: a retrospective medical record review

**DOI:** 10.1186/s12883-016-0698-9

**Published:** 2016-09-29

**Authors:** Tara Nazareth, Howard S. Friedman, Prakash Navaratnam, Denise A. Herriott, John J. Ko, Peri Barr, Rahul Sasane

**Affiliations:** 1 Novartis Pharmaceutical Corporation, East Hanover, NJ USA; 2 DataMed Solutions LLC, New York, NY USA; 3 Indegene TTM, Atlanta, GA USA

**Keywords:** Disease-modifying therapy, Oral, Multiple sclerosis, Retrospective, Medical record, Resource use, Prescribing, Dimethyl fumarate, Fingolimod, Teriflunomide, Persistency

## Abstract

**Background:**

In the US, the approved multiple sclerosis (MS) oral disease-modifying therapies (ODMTs) are fingolimod (FTY), teriflunomide (TFN), and dimethyl fumarate (DMF). FTY and TFN are recommended with once-daily doses with no up-titration, whereas DMF treatment is recommended twice-daily (BID) and is initiated with a 7-day starter dose of 120 mg BID before up-titration to the maintenance dose of 240 mg BID. Limited information exists regarding real-world ODMT prescribing patterns to aid physician/patient decision-making.

**Methods:**

Eligible patients for this retrospective medical record review were ≥18 years, had one visit related to ODMT initiation (index visit), and ≥1 visit within 12 months before and after the index visit. Primary objectives were to assess post-index ODMT persistency (i.e., discontinuation), prescribing patterns (medication switching, dose up-titrations, dose reduction, re-starts, and add-ons) and medical resource utilization (office-visits, MRI procedures, and mobility indicators) at distinct time windows of 3, 6, 9, and 12 months. Chi-square or Wilcoxon Rank Sum tests were used for 3-way ODMT group comparisons.

**Results:**

Medical records of 293 MS-diagnosed patients using ODMTs were abstracted from 19 US-based neurology clinics between December 31, 2010 and June 30, 2014 (FTY: 101; DMF: 133; TFN: 59). Persistency rates among ODMT groups were similar. MS-related medication switching, dose reduction, re-starts, and add-ons were infrequently observed and were similar across ODMT groups. Of DMF patients with a confirmed starting dose of 120 mg BID with ≥12 months follow-up (*n* = 26), the percentage who were prescribed dose up-titrations to the recommended maintenance DMF dose was 23.1 % at 1–3 months, 26.9 % at 4–6 months, 42.3 % at 7–9 months, and 0 % at 10–12 months. There were no significant differences at any time window among the ODMT groups in the number of office visits or percent of patients receiving MRIs. Mobility indicator patterns (proportion of patients with abnormal gait, wheelchair use, etc.) were consistent over time.

**Conclusions:**

There was no difference in persistency, prescribing patterns (medication switching, dose reduction, re-starts, and add-ons) or medical resource utilization (office-visits, MRI procedures, and mobility indicators) among the ODMTs. However, in a small sub-group of patients, delays of up to 9 months in DMF dose-up titration to the recommended maintenance dose were observed.

**Electronic supplementary material:**

The online version of this article (doi:10.1186/s12883-016-0698-9) contains supplementary material, which is available to authorized users.

## Background

Disease modifying therapies (DMTs) in relapsing-remitting multiple sclerosis (RRMS) have been shown to prevent relapses and reduce the magnetic resonance imaging (MRI)-apparent lesions that are indicative of MS activity [1]. Until 2010, only injectable DMTs were available for patients with RRMS. The proportion of patients adherent to injectable DMTs ranges from approximately 55 to 90 % [2, 3] and persistence after 1 year is approximately 50 % [4]. In a systematic review of observational and randomized, controlled trials of injectable DMTs, the most-cited reasons for discontinuing therapy were adverse events and lack of efficacy [5]. Furthermore, data from the review revealed a high incidence of flu-like symptoms and injection site reactions that persisted over time. Such tolerability issues have been shown to directly impact persistence and adherence [6, 7].

As of 2015, three oral DMTs (ODMTs) [i.e., fingolimod (FTY), teriflunomide (TFN), and dimethyl fumarate (DMF)] have become available in many countries, including the US, as an alternative to injectable DMTs. In randomized, double-blind, placebo-controlled trials FTY, TFN, and DMF significantly reduced the annualized relapse rate and the occurrence of MRI lesions when compared with placebo [8–10].

The three ODMTs differ in how they are dosed in patients with RRMS. DMF is available as a starter dose (120 mg twice daily [BID]) and a maintenance dose (240 mg BID), with the dose titration to 240 mg BID recommended after the first 7 days of use [11]. For patients who do not tolerate the DMF maintenance dose, temporary dose reductions to 120 mg BID may be considered, but the full maintenance dose should be resumed within 4 weeks. In contrast, FTY is only available in one dose (0.5 mg), and TFN is available in two doses (14 mg and 7 mg); neither have starter doses in their regimens [12, 13]. DMF is taken twice a day (both starter dose and maintenance dose), whereas FTY and TFN are taken once a day. Only patients taking FTY are required to have a first dose observation involving a baseline electrocardiogram (ECG) and a ≥6 h observation period.

Given the availability of multiple DMTs, and that several ODMTs are relatively new to the US market, information regarding real-world ODMT prescribing patterns is important for appropriate prescribing of these agents. Information on the characteristics and healthcare use of patients receiving each ODMT could provide clues as to prescriber-perceived efficacy and safety of ODMTs, as well as provide basic information on healthcare resource burden. Thus, the primary objective of this analysis was to describe the MS-related medication persistency, prescribing patterns, and concomitant medical resource utilization of patients with MS receiving ODMTs in the US. Secondary objectives were to describe the demographics and clinical characteristics of US patients prescribed ODMTs.

## Methods

### Study overview

In this retrospective medical record abstraction study, a sample of MS patients was drawn from a target goal of 25 specialty neurology practices. The practices were pre-identified as high prescribers (≥10 prescriptions from March 2013 to March 2014) of ODMT agents using data obtained from IMS Health. In order to reflect US disease prevalence and treatment patterns, geographic distribution of site selection in this study was modelled according to US region per the distribution of prescribers observed in the IMS Health data, such that if 50 % of prescribers were in the South according to the IMS Health data, 50 % of the study sample’s prescribers enrolled for this study were from the South. The goal was 250 medical charts in a 2:2:1 ratio for FTY, DMF, and TFN; the ratio was determined based on market share at the time of study planning. Physicians at the sites were asked to contribute charts based on inclusion and exclusion criteria. A minimum of five charts and a maximum of 15 charts was collected from participating sites. Charts were non-randomly reviewed on a first-served basis until the quota of charts was reached for each drug. A request was submitted and granted by the reviewing institutional review board waiving the requirement for patient informed consent; approval to conduct the study was obtained from a centralized institutional review board (Sterling IRB; Atlanta, GA).

An identification period from December 31, 2011 to December 31, 2013 was used to identify the first record of use of an ODMT (index date) via prescription or chart notation. The total observation period of the study was from December 31, 2010 to June 30, 2014. Eligible patients were ≥18 years of age on the index date, had at least one record of a physician-prescribed ODMT during the identification period of the study, had at least one visit to the site neurology practice in the 12 months before the index date (pre-index period, baseline visit; anytime between December 31, 2010 and December 31, 2012), and had at least one additional site visit in the post-index period (in addition to the index date visit; anytime between December 31, 2012 and June 30, 2014). Patients with missing documentation of MS diagnosis or missing information on sex and/or age in their medical record were excluded from the study.

### Record abstraction process

The abstractors used in this study were nurses or pharmacists with previous experience abstracting information from neurology or MS patient records. To help ensure consistency of data collection, the abstractors were trained on the study design and presented with standardized data collection forms and a detailed codebook which contained definitions of the target variables. They were also trained on mandatory safety reporting procedures as required by the study sponsor.

Upon completion of the data collection process, a quality control assessment was completed by study investigators to ensure adequate data capture and quality. Each abstractor’s responses underwent an inter-rater reliability check to ensure consistency.

De-identified data were captured on a paper optical character recognition form that was scanned into a Microsoft Access database. From there, it was imported into SAS version 9.2 and checked for appropriate values using logic and range checks by the record abstraction research manager.

### Information collected from medical records

Information collected during the study period from each record included demographics, clinical characteristics (i.e., MS diagnosis, year of diagnosis, comorbidities, MS symptoms, etc.), ODMT prescriptions, symptomatic medication prescriptions, and medical resource utilization. Information was collected on ODMT persistency (proportion of patients discontinuing treatment) and prescribing patterns in the post-index period based on a review of doses, and/or physician narratives; patterns assessed included dose titration to recommended maintenance doses, dose reductions, switching to another MS DMT, re-starts (re-initiation of index ODMT ≥30 days after discontinuation), and add-ons (treatment with additional MS DMTs with no indication the index ODMT was discontinued). Post-index medical resource utilization was also extracted, including the number of outpatient office visits and MRI procedures. Notations of mobility indicators (abnormal gait, dizzy gait, walk trials [e.g., timed 25-ft walk trial], wheelchair use, and crutch/cane/walker use) were tracked post-index. An additional excel file shows the raw data collected in detail (see Additional file 1).

### Data analysis

All post-index abstracted information was explored in a cumulative fashion at distinct time windows of 3, 6, 9, and 12 months. Patients had variable enrollment lengths; thus, the patient sample size was different for each post-index time period (Table 1). An additional analysis for dose titration to recommended maintenance doses for DMF was conducted among the subset of DMF patients who had a confirmed starting dose of 120 mg BID. Furthermore, dose titration analyses were conducted at each post-index time period using only the subset of patients with a follow-up of at least 9 or 12 months to gain longitudinal insight into observed up-titrations.

**Table 1:** Patient followed up/follow-up information

	Total	FTY	DMF	TFN	*P*-value (3 groups)
	(*N* = 293)	(*N* = 101)	(*N* = 133)	(*N* = 59)	
Index date, (SD)	7/11/2013 (180 days)	6/6/2013 (235 days)	8/15/2013^a^ (128 days)	6/29/2013 (159 days)	0.0370
Enrollment end date, (SD)	7/4/2014 (17 days)	7/5/2014 (17 days)	7/3/2014 (16 days)	7/5/2014 (20 days)	0.1326
Mean follow-up duration, days (SD)	357.6 (176.5)	393.5 (229.8)	323.0 (123.6)	371.3 (156.9)	0.0300
Patients enrolled at least X months, *n* (%)					
3 months	281 (95.9)	98 (97.0)	124 (93.2)	59 (100)	0.0720
6 months	236 (80.5)	79 (78.2)	106 (79.7)	51 (86.4)	0.4235
9 months	188 (64.2)	63 (62.3)	85 (63.9)	40 (67.8)	0.7856
12 months	151 (51.5)	55 (54.5)	65 (48.9)	31 (52.5)	0.6885

*DMF* dimethyl fumarate, *FTY* fingolimod, *TFN* teriflunomide

^a^ *n* = 128

Both descriptive and univariate statistical analyses designed to compare pre-index (baseline), index, and post-index abstracted variables between the ODMT patient cohorts were conducted. Only reported values were analyzed; no imputation was made for missing data since it was not possible to determine if a missing observation was due to no information or an oversight in recording. All categorical variables were reported as raw counts or percentages and were analyzed using Chi-square tests. For the Chi-square tests, test results were deemed ‘indeterminant’ when 50 % of more of the cells compared had less than five observations. Continuous variables were analyzed using the non-parametric Wilcoxon Rank Sum test. Two sided *p*-values <0.05 denoting statistical significance were reported where applicable. All analyses were conducted using SAS version 9.3 statistical software (Cary, NC).

## Results

### Patient demographics and clinical characteristics

**Fig. 1:**
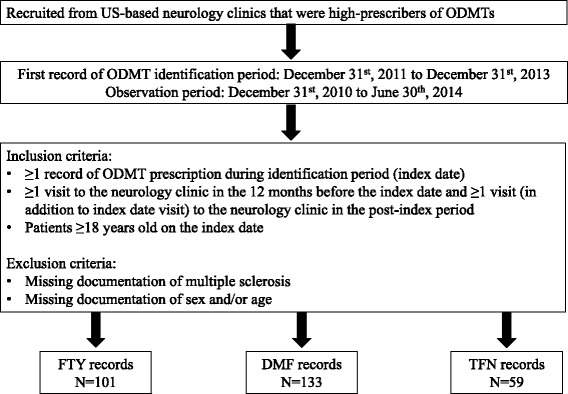
Medical record selection process. DMF = dimethyl fumarate; ODMT = oral disease-modifying therapy; FTY = fingolimod; TFN = teriflunomide

Medical records of 293 MS-diagnosed patients using ODMTs were abstracted from 19 US-based neurology clinics between December 31, 2010 and June 30, 2014 (Fig. 1). Of the 293 records, 101 were for FTY, 133 were for DMF, and 59 were for TFN. The patients prescribed FTY were younger than the DMF and TFN patients (mean ± SD; 44.2 ± 10.7 years vs 49.6 ± 10.3 years vs 50.6 ± 9.6 years, respectively; 3-way comparison *P* < 0.0001; Table 2), and had a longer period of follow-up (393.5 days vs 323.0 days vs 371.3 days, respectively; 3-way comparison *P* = 0.0300; Table 1). There was a significant difference in the regional distribution of patients with prescriptions for ODMTs in this study sample (3-way comparison *P* < 0.0001). A large proportion of patients prescribed FTY and TFN resided in the South (>40 %), whereas only 1 % of the patients prescribed FTY resided in the West (Table 2).

**Table 2:** Demographics of ODMT patients

	Total	FTY	DMF	TFN	*P*-value (3 groups)
	(*N* = 293)	(*N* = 101)	(*N* = 133)	(*N* = 59)	
Female, *n* (%)	214 (73.0)	75 (74.3)	96 (72.2)	43 (72.9)	0.9386
Mean age, y (SD)	48.0 (10.7)	44.2 (10.7)	49.6 (10.3)	50.6 (9.6)	<0.0001
Age group, *n* (%)					0.0349
18–24 years	2 (0.7)	2 (2.0)	0	0	
25–34 years	33 (11.3)	18 (17.8)	12 (9.1)	3 (5.1)	
35–44 years	81 (27.7)	34 (33.7)	32 (24.2)	15 (25.4)	
45–54 years	100 (34.3)	31 (30.7)	49 (37.1)	20 (33.9)	
55–64 years	59 (20.2)	12 (11.9)	29 (22.0)	18 (30.5)	
65–74 years	13 (4.5)	4 (4.0)	7 (5.3)	2 (3.4)	
75+ years	4 (1.4)	0	3 (2.3)	1 (1.7)	
Missing	1 (0.3)	0	0	0	
Region, *n* (%)					<0.0001
Midwest	73 (25.1)	32 (32.3)	37 (27.8)	4 (6.8)	
Northeast	58 (19.9)	24 (24.2)	26 (19.6)	8 (13.6)	
South	105 (36.1)	42 (42.4)	37 (27.8)	26 (44.1)	
West	55 (18.9)	1 (1.0)	33 (24.8)	21 (35.6)	
Missing	2 (0.7)	0	0	0	
Race, *n* (%)					0.0074
White	61 (21.1)	29 (28.7)	28 (21.5)	4 (6.9)	
Black	11 (3.8)	7 (6.9)	1 (0.8)	3 (5.2)	
Other	3 (1.0)	1 (1.0)	1 (0.8)	1 (1.7)	
Unknown	214 (74.1)	64 (63.4)	100 (76.9)	50 (86.2)	
Missing	4 (1.4)	0	0	0	
Insurance, *n* (%)					0.0051
Commercial	70 (24.1)	15 (15.0)	35 (26.5)	20 (34.5)	
Medicaid	8 (2.8)	0	6 (4.6)	2 (3.5)	
Medicare	28 (9.7)	4 (4.0)	16 (12.1)	8 (13.8)	
Cash	2 (0.7)	1 (1.0)	1 (0.8)	0	
Other	5 (1.7)	2 (2.0)	3 (2.3)	0	
Unknown	177 (61.0)	78 (78.0)	71 (53.8)	28 (48.3)	
Missing	3 (1.0)	0	0	0	

*DMF* dimethyl fumarate, *ODMT* oral disease-modifying therapy, *FTY* fingolimod, *TFN* teriflunomide

The vast majority of comorbidities pre-specified for examination in the study, such as heart disease, asthma, stroke, arthralgia, etc., were not observed in medical records (documented in less than 2 % of patients); therefore, statistical analysis was limited to depression, diabetes, and hypertension. Patients prescribed FTY had a lower incidence of reported hypertension compared with DMF and TFN (3.0 vs 11.3 vs 18.6 %, respectively; 3-way comparison *P* = 0.00461); however, in general there did not appear to be any distinct comorbidity patterns across the ODMT patient groups.

**Fig. 2:**
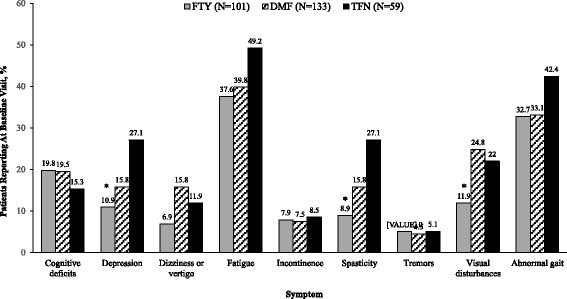
Incidence of reported multiple sclerosis symptoms at the baseline visit. *P ≤ 0.05 for 3-way comparison. DMF = dimethyl fumarate; FTY = fingolimod; TFN = teriflunomide

At the baseline visit, fatigue and abnormal gait were the most commonly reported MS symptoms (Fig. 2). The incidence of spasticity, depression, and visual disturbances was significantly different among the three ODMT patient groups and was lowest in patients prescribed FTY when compared with DMF and TFN (3-way comparison *P* ≤ 0.05; Fig. 2). In the post-index follow-up time windows of 3, 6, 9, and 12 months, there were no consistent patterns in the differences in MS symptoms among the ODMT patient groups.

**Fig. 3:**
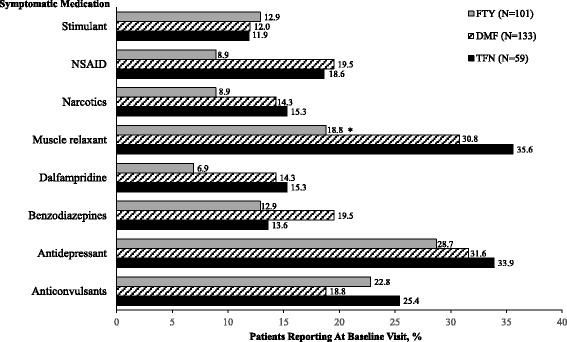
Incidence of reported symptomatic medication use at the baseline visit. Only medication use with an incidence of ≥10 % in any group is reported. *P = 0.04 for 3-way comparison. DMF = dimethyl fumarate; FTY = fingolimod; NSAID = non-steroidal anti-inflammatory drug; TFN = teriflunomide

At the baseline visit, antidepressants and muscle relaxants were the most common symptomatic drug prescriptions (Fig. 3). The percentage of patients with muscle relaxant prescriptions was significantly different among the three ODMT patient groups, and was lowest in patients prescribed FTY compared with DMF and TFN (3-way comparison *P* = 0.04; Fig. 3). In the post-index follow-up time windows of 3, 6, 9, and 12 months, symptomatic drug prescriptions were not often reported.

### ODMT persistency and prescribing patterns

**Fig. 4:**
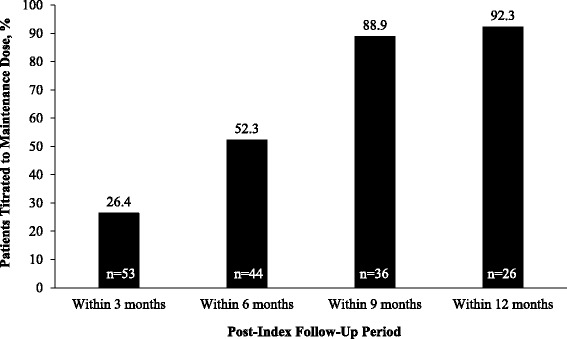
Cumulative percentage of patients prescribed DMF who had a confirmed starting dose of 120 mg twice-daily, and who were prescribed a dose up-titration to the recommended maintenance dose. DMF = dimethyl fumarate

**Fig. 5:**
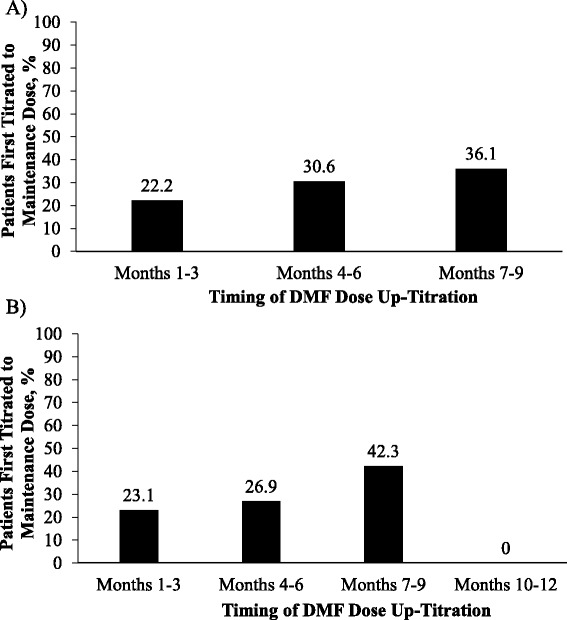
Timing of first DMF dose titrations to the recommended maintenance dose in patients with a confirmed starting dose of 120 mg twice-daily, and followed-up for at least **a** 9 months (*n* = 36) or **b** 12 months (*n* = 26). DMF = dimethyl fumarate

In the post-index period, ODMT persistency and prescribing patterns were tracked at each distinct time window in a cumulative fashion at 3, 6, 9, and 12 months to assess change over time (Table 3). Because patients had varying durations in their time of follow-up, the size of the patient samples was different for each time window. There was no significant difference in the persistency rate among the ODMT patient groups at any time window (Table 3); the overall rate of discontinuation (%) within 3, 6, 9, and 12 months was 4.3, 7.6, 13.3, and 14.6, respectively. MS-related medication switching, ODMT dose reduction, ODMT re-starting, and add-on treatments were observed infrequently (each outcome identified in a maximum of 4 % of patients within 9 months and 7 % of patients within 12 months), and were similar across the ODMT patient groups. The frequency of dose titrations to recommended maintenance doses with DMF was assessed at each post-index time window in patients that had a confirmed starting dose of 120 mg BID. This analysis revealed that by 3, 6, 9, and 12 months, a cumulative total of 26.4, 52.3, 88.9, and 92.3 % of patients, respectively, had a recorded dose titration to the recommended maintenance DMF dose (Fig. 4). A longitudinal analysis tracking DMF patients with a confirmed starting dose of 120 mg BID, and who had follow-up durations of at least 9 months (*n* = 36 of 133 DMF patients) or 12 months (*n* = 26 of 133 DMF patients), revealed delays in the dose titration to the recommended maintenance dose (Fig. 5). The proportion of DMF patients with ≥9 months follow-up who experienced their first dose titration to the recommended maintenance dose was 22.2 % at 1–3 months, 30.6 % at 4–6 months, and 36.1 % at 7–9 months, and in patients with ≥12 months follow-up was 23.1 % at 1–3 months, 26.9 % at 4–6 months, and 42.3 % at 7–9 months (0 % at 10–12 months).

**Table 3:** ODMT discontinuation rates at cumulative post-index time windows of within 3, 6, 9, and 12 months. Because patients had varying durations in their time of follow-up, sample sizes were different for each time window. *P*-values for medication switching, dose reduction, re-starts, and add-ons were indeterminate, therefore, these data are not shown

Prescribing parameters, *n* (%)	Total	FTY	DMF	TFN	*P*-value (3 groups)
	(*N* = 293)	(*N* = 101)	(*N* = 133)	(*N* = 59)	
Within 3 months					
Patients, *n*	281	98	124	59	
Discontinuation	12 (4.3)	5 (5.1)	5 (4)	2 (3.4)	0.8629
Within 6 months					
Patients, *n*	236	79	106	51	
Discontinuation	18 (7.6)	5 (6.3)	6 (5.7)	7 (13.7)	0.177
Within 9 months					
Patients, *n*	188	63	85	40	
Discontinuation	25 (13.3)	6 (9.5)	12 (14.1)	7 (17.5)	0.4866
Within 12 months					
Patients, *n*	151	55	65	31	
Discontinuation	22 (14.6)	6 (10.9)	9 (13.8)	7 (22.6)	0.3299

*DMF* dimethyl fumarate, *ODMT* oral disease-modifying therapy, *FTY* fingolimod, *TFN* teriflunomide

### Medical resource utilization

In the post-index period, there were no significant differences at any time window among the ODMT patient groups in the number of outpatient office visits (range of 2.3 to 2.6 visits within 12 months), or percent of patients receiving MRIs (range of 32.3 to 49.2 % within 12 months). At the 3-month post-index time window, the most commonly recorded mobility indicator for all three ODMT patient groups was abnormal gait (31.0 %); the least commonly recorded mobility indicator was wheelchair use (4.6 %). Walking with a crutch/cane/walker was significantly different among the ODMT groups, and was highest in patients prescribed TFN (18.6 %; 3-way comparison *P* = 0.05). The mobility indicator patterns remained consistent over the 6, 9, and 12 month time windows; however, the statistical difference for walking with a crutch/cane/walker disappeared, and there were no other significant differences among the ODMT patient groups.

## Discussion

Analysis of ODMT discontinuations in this study indicated that the persistency rate did not differ among ODMT patient groups FTY, DMF, and TFN. Reports of MS-related medication switching, ODMT dose reduction, ODMT re-starting, and add-on treatments were infrequent. Delays in the up-titration to the maintenance dose of DMF were observed up to 9 months after treatment initiation.

Assessment of medical resource utilization among the ODMT patient groups revealed no notable differences in the number of outpatient office visits, MRI procedures, or mobility patterns. When examining demographics and clinical characteristics of patients prescribed ODMTs, patients prescribed FTY were on average younger and had a longer follow-up period than patients prescribed DMF or TFN. The comorbidity profile appeared to be similar between the ODMT cohorts with the exception of hypertension. The lower incidence of hypertension with FTY may be due to cautious prescribing to patients with cardiac conditions per prescribing information.

The lack and/or delay of a recorded dose up-titration to the maintenance dose observed for many of the patients prescribed DMF is an important finding within our examination of real-world ODMT use. Per the FDA prescribing information, the initial 120 mg BID dose should be taken for 7 days, followed by dose escalation to the maintenance 240 mg BID dose [11]. There are no efficacy data to support use of the 120 mg BID DMF dose as a maintenance dose [14]. Therefore, it is possible patients who experience delays up-titrating to the DMF maintenance dose beyond the recommended 7-day initial dosing period may be more likely to experience negative outcomes such as relapses or disease progression. More research is needed to understand the extent of delays and the impact of under-dosing patterns associated with DMF, as observed in this study.

The reasons for the delay in DMF up-titration are unknown. One possibility is that DMF is a relatively new DMT, and cautionary prescribing could result in some delays in up-titration, although this is unlikely to account for the length of the delays observed in this study. Another possibility is a delay due to concerns regarding gastrointestinal intolerance with the maintenance dose as observed in clinical trials. In pivotal clinical trials for DMF, gastrointestinal adverse events (i.e. diarrhea, nausea, upper abdominal pain, vomiting) occurred in a combined total of 42 % of subjects receiving DMF, and a total of 4 % of subjects discontinued due to gastrointestinal events [8, 15, 16].

Persistence of medication use is a major hurdle for the treatment of chronic disease. Of the total 151 patients with 12 months of follow-up in this analysis, the rate of discontinuation overall with the ODMTs was 14.6 %. The rates observed in this study were lower than the discontinuation rates reported in other “real world” retrospective analyses of ODMTs. However, differences are expected due to variation in data source and study design. In one retrospective claims database analysis of 3,750 US MS patients, discontinuation within 1 year of initiation was 27.9 % for FTY, 39.5 % for glatiramer acetate, 43.7 % for interferon, and 39.0 % for natalizumab [2]. This FTY discontinuation rate is nearly identical to that found in another retrospective claims analysis of 1,891 US MS patients in a different database, where the rate of discontinuation for FTY after 1 year was 27.8 % [4]. One-year discontinuation rates for DMF and TFN outside of clinical trials have yet to be investigated; however, shorter-term analyses have found differences in discontinuation among oral DMTs in smaller timeframes. A single-center analysis of 743 MS patients initiating FTY or DMF found that the 3-month discontinuation rates were significantly different at 8 and 16 %, respectively [17]. The results from the current analysis did not reveal any significant difference in discontinuation rates among the three ODMTs.

The strength of this analysis is that it is the first to evaluate real world of all the available ODMTs in one study with up to a 1-year follow-up time frame. Medical records provide a unique insight into health care providers’ view of the MS patient and ODMT as well as richer clinical detail on patients’ conditions and care. Chart abstraction studies are known to be subject to documentation practices and data capture limitations, which may impact medication prescribing insights as well as insights into patient characteristics. The MS symptoms were captured as reported in the medical charts and were not necessarily defined by specific criteria (e.g., Diagnostic and Statistical Manual of Mental Disorders to diagnose depression); therefore, symptom results must be interpreted with caution. There may also have been a lag in recording of events, such that the timing of the prescribing patterns reported here may only be reflective of the time the events were recorded and not when the actual events occurred. However, it is very likely that these prescribing patterns coincided with formal care-seeking behavior, such as office visits. Furthermore, medication utilization as notated in the chart denotes what the physician prescribed and does not account for subsequent patient behavior. It is also possible that patients received medication samples from their prescribing physician, which may or may not have been captured in the medical record. Our study represents a convenience sample and may be subject to participation bias. Lastly, the length of follow-up was 12 months and the number of patients with 12-month follow-up data in every ODMT group was small; replication of the study with a longer follow-up and more patients may be warranted to examine prescribing patterns, healthcare resource utilization and patient types, and determine the impact on disease progression, relapse rates, and mobility indicators in MS.

## Conclusions

Our medical chart abstraction study found no difference in prescribing patterns (i.e. discontinuation, medication switching, dose reduction, re-starts, and add-ons) or medical resource utilization (office-visits, MRI procedures, and mobility indicators) among the ODMTs. However, in a small sub-group of patients who started on the 120 mg BID dose, delays of up to 9 months in DMF dose-up titration to the recommended maintenance dose were observed. Patients who experience these delays may be more likely to experience negative outcomes. Further study with more patients and longer follow-up is warranted to confirm and elaborate upon our findings.

## Additional file

Additional file 1: Multiple sclerosis patients receiving oral disease-modifying therapies medical record review. Data file contains multiple-sclerosis patient medical record reviews. Each row is a distinct patient. Columns reflect information about the patient including baseline information as well as information specific to clinical visits. Subscripts in the field name found in line 1 ("_2", "_3", etc.) reflect the visit number. (XLSX 3109 kb)

## Abbreviations

BID: Twice daily
DMF: Dimethyl fumarate
FTY: Fingolimod
MRI: Magnetic resonance imaging
MS: Multiple sclerosis
ODMT: Oral disease-modifying therapy
RRMS: Relapsing-remitting multiple sclerosis
TFN: Teriflunomide
